# Prevalence of asymptomatic strongyloidiasis co-infection in COVID-19 patients residing in endemic areas

**DOI:** 10.1186/s40001-023-01262-9

**Published:** 2023-08-11

**Authors:** Alireza Ashiri, Molouk Beiromvand, Abdollah Rafiei, Reza Heidari, Ameneh Takesh

**Affiliations:** 1 Student Research Committee, Ahvaz Jundishapur University of Medical Sciences, Ahvaz, Iran; 2 Department of Parasitology, School of Medicine, Ahvaz Jundishapur University of Medical Sciences, Ahvaz, Iran; 3 Infectious and Tropical Diseases Research Center, Health Research Institute, Ahvaz Jundishapur University of Medical Sciences, Ahvaz, Iran; 4 Department of Microbiology, Faculty of Medicine, Ahvaz Jundishapur University of Medical Sciences, Ahvaz, Iran; 5 Department of Medical Mycology, School of Medicine, Ahvaz Jundishapur University of Medical Sciences, Ahvaz, Iran

**Keywords:** Strongyloidiasis, COVID-19, Eosinophilia, ELISA, Khuzestan Province, Iran

## Abstract

**Background:**

Fatal forms of strongyloidiasis, hyperinfection syndrome (HS) and disseminated strongyloidiasis (DS), are caused by exaggerated autoinfection of the intestinal nematode, *Strongyloides stercoralis* (*S. stercoralis*). Corticosteroids, frequently administered to patients with severe COVID-19, can transform chronic asymptomatic strongyloidiasis into the above-mentioned fatal diseases. This study aimed to investigate the prevalence of strongyloidiasis in COVID-19 patients receiving corticosteroids in a hypoendemic region.

**Methods:**

The present cross-sectional study enrolled 308 COVID-19 patients admitted to two hospitals in Ahvaz and Abadan in the southwest of Iran between 2020 and 2022. A real-time reverse transcription polymerase chain reaction (RT-PCR) test and chest computed tomography (CT) scan were employed to detect and monitor the disease’s severity in the patients, respectively. All patients were evaluated for IgG/IgM against *S. stercoralis* using Enzyme-linked immunosorbent assay (ELISA) test. Subsequently, individuals with a positive ELISA test were confirmed using parasitological methods, including direct smear and agar plate culture (APC).

**Results:**

The patients were between 15 and 94 years old, with a mean age of 57.99 ± 17.4 years. Of the 308 patients, 12 (3.9%) had a positive ELISA test, while 296 (96.1%) had negative results. Three of the 12 patients with a positive ELISA result died, and three failed to provide a stool sample. To this end, only six cases were examined parasitologically, in which *S. stercoralis* larvae were observed in five patients. Significant differences were found between *S. stercoralis* infection with sex (*p* = 0.037) and age (*p* = 0.027). Binary regression analysis revealed that strongyloidiasis was positively associated with sex (odds ratio [OR]: 5.137; 95% confidence interval [CI]: 1.107–23.847), age (OR: 5.647; 95% CI 1.216–26.218), and location (OR: 3.254; 95% CI: 0.864–12.257).

**Conclusions:**

Our findings suggest that screening for latent strongyloidiasis in COVID-19 patients in endemic areas using high-sensitivity diagnostic methods, particularly ELISA, before receiving suppressive drugs should be given more consideration.

## Background

*Strongyloides stercoralis* (*S. stercoralis*), a soil-borne intestinal nematode, has a unique life cycle with the potential of autoinfection. The parasite-induced infection can range from asymptomatic chronic infection to fatal disseminated infection. Evidence suggests that immunosuppression caused by corticosteroid use, one of the main risk factors, can lead to uncontrolled proliferation of the nematode in asymptomatic patients, resulting in severe strongyloidiasis, hyperinfection syndrome/ disseminated syndrome (HS/DS), with mortality rate of up to 100% [1, 2]. On the other hand, chronic strongyloidiasis is frequently misdiagnosed due to the low sensitivity of routine parasite diagnostic tests, such as direct smear examination [3]. Thus, diagnosing the infection is typically challenging before and during immunosuppression [4]. Serial stool examinations, culturing on nutrient agar plate, or searching for specific antibodies in the serum using the enzyme-linked immunosorbent assay (ELISA) technique can increase the likelihood of detecting these cases [5, 6].

Coronavirus disease 2019 (COVID-19) is a respiratory disease induced by severe acute respiratory syndrome coronavirus 2 (SARS-CoV-2). Diagnosis of the disease is mainly based on viral nucleic acid detection using reverse transcription polymerase chain reaction (RT-PCR) and chest computed tomography (CT) [7]. The overproduction of the pro-inflammatory cytokines, also known as cytokine storm syndrome (CSS), is an important manifestation of critical COVID-19 [8]. Patients with moderate or severe infections are often prescribed cytokine inhibitors, such as tocilizumab (TCZ), and the corticosteroid dexamethasone due to the tissue damage caused by this phenomenon, particularly in the respiratory system [8–11]. Using these medications in patients co-infected with SARS-CoV-2, and *S. stercoralis* can reactivate latent strongyloidiasis [9]. Several cases of severe strongyloidiasis in natives of or recent arrivals to endemic areas were reported during the COVID-19 pandemic [12–15].

Considering the background provided above, screening for strongyloidiasis in COVID-19 patients, increasing public health system and healthcare provider awareness, and refining diagnostic approaches for *S. stercoralis* detection are essential [16].

Iran is a hypoendemic area for strongyloidiasis, with a prevalence rate of 4.8% (2.5–7.2%) [17]. In immunocompetent and immunocompromised Iranian populations, the prevalence of strongyloidiasis has been estimated to be 2% and 4%, respectively [18]. Due to the region’s humid and tropical climate, the prevalence of strongyloidiasis in Khuzestan province is 8.7% using serological methods and 2.7% using parasitological methods [3]. In addition, Khuzestan was severely impacted by the COVID-19 pandemic and was highly prevalent among patients [19]. As a result, the current study aimed to determine the prevalence of *S. stercoralis* infection in COVID-19 patients admitted to the province's two regional hospitals.

## Methods

### Study location

With a population of over 4,700,000 and an area spanning 63,238 Km^2^ [19], Khuzestan province is one of the most important agricultural and industrial regions in southwestern Iran [20]. Due to the region’s hot and humid climate, most neglected tropical diseases (NTDs) may be endemic in the area [3]. To this end, the current study was conducted in two of Iran’s most populous counties, Ahvaz and Abadan.

### Study design and sampling

During multiple peaks of the coronavirus pandemic from 2020 to 2022, this cross-sectional study was conducted on patients hospitalized with moderate to severe COVID-19 in Ahvaz and Abadan counties, southwestern Iran, where strongyloidiasis is endemic. The inclusion criteria were hospitalized patients with COVID-19 who had a fever, headache, sore throat, cough, chills, rhinorrhea, and respiratory distress and whose infection with SARS-CoV-2 was confirmed using an RT-PCR test on nasopharyngeal swabs. A chest CT scan was utilized to assess the severity of the disease. According to their electronic medical records, all patients had received dexamethasone and tocilizumab. Patients who received antiparasitic medications within the previous six months were excluded from the study.

The required sample size was determined using single population proportion formula: (Zα/_2_)^2^ × *p* × (1 − p)/d2, where n is the sample size, z is the standard normal score set at 1.96, d is the desired degree of accuracy and p is the estimated proportion of the target population. By taking a prevalence rate of 8.7% [3], Zα/2 = 1.96 and 95% confidence interval, and d = 0.033 the computed sample size was 305. Using simple random sampling, finally 308 patients, including 157 from Ahvaz and 151 from Abadan, were selected and included in the study. Twenty cases were randomly selected each week, and five patients were enrolled in the study using a lottery system based on the inclusion criteria.

### Data collection

A questionnaire was used to collect demographic data such as age, sex, and place of residence, but clinical and laboratory findings, as well as information about medications used, were obtained from the hospital’s electronic database.

### Enzyme-linked immunosorbent assay (ELISA)

To determine the prevalence of *S. stercoralis*/SARS-CoV-2 co-infections, all COVID-19 patients were screened for *S. stercoralis* infection using ELISA test. Each patient had 2 mL of venous blood drawn and centrifuged at 2000×g for 5 min; the isolated sera were stored at − 20 °C until ELISA testing. The positive and equivocal ELISA test patients were subsequently examined using parasitological techniques, such as direct smear and agar plate culture (APC), for additional confirmation.

The test was conducted using an IgG/IgM ELISA kit (NovaTec Immundiagnostica GmbH, Dietzenbach, Germany) according to the manufacturer’s instructions. The plate was coated with recombinant immunodiagnostic antigens (NIE) that had a diagnostic sensitivity and specificity of 89.47% (95% CI: 75.2–97.06%) and 94.12% (95% CI: 83.76–98.77%) respectively. A DS2^®^ ELISA reader (Dynex, VA, USA) measured optical density (OD) at 450 nm. The cut-off point was set at 11 NovaTec Units (NTU). A 9–11 NTU range was considered equivocal, while less than 9 NTU was considered negative.

### Direct smear examination and agar plate culture (APC)

All patients who tested positive for strongyloidiasis or were suspected of having the disease based on ELISA results were asked to provide stool samples. Each patient provided two stool samples on separate days, which were analyzed by direct smear and APC to confirm the ELISA results. Four slides were prepared from each sample for direct smear examinations and examined at low magnification to increase the sensitivity of larva detection. APC was determined by placing 3–4 g of fresh stool sample on the center of a nutrient agar plate. The plates’ lids were then sealed to prevent the larvae from escaping. The plates were then placed in a plastic box and incubated for 1 week at 25–30 °C.

**Fig. 1:**
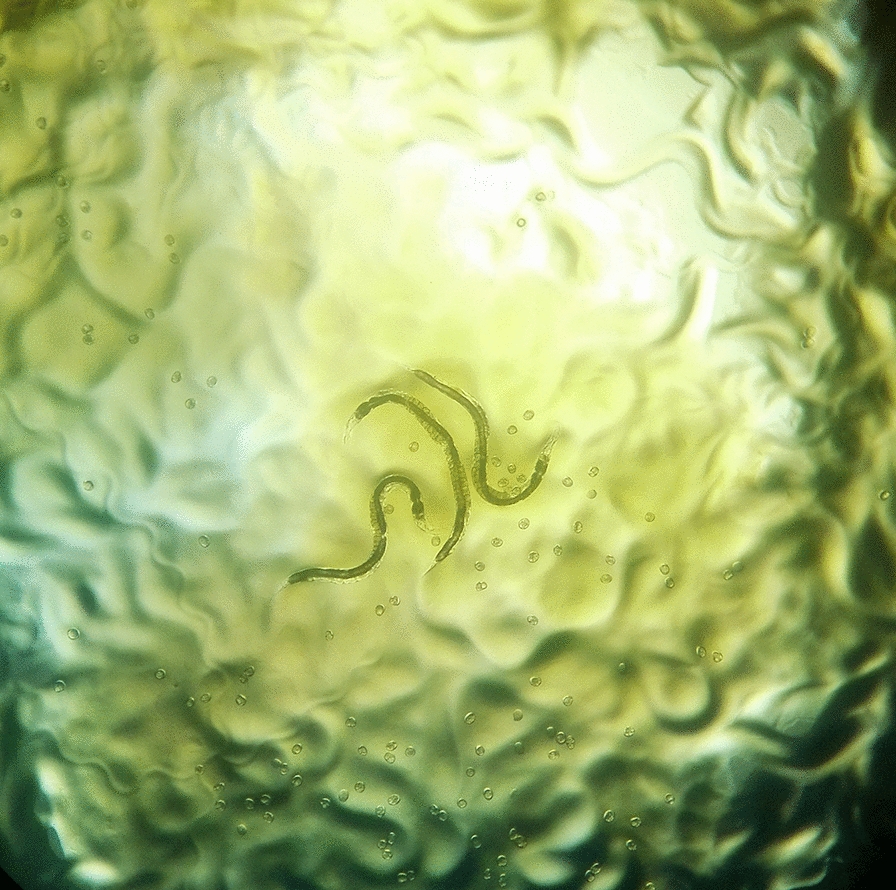
*Strongyloides stercoralis* adult worms, ova, and larva’ tracks on agar plate culture

**Fig. 2:**
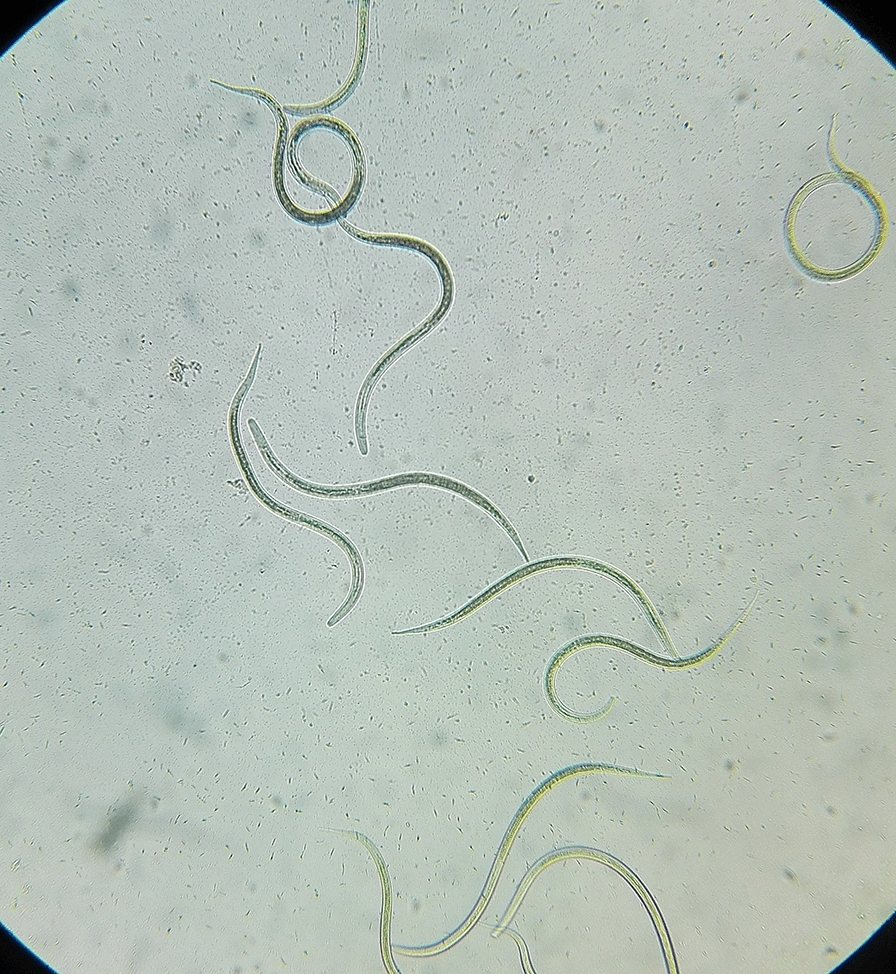
Filariform larva of *Strongyloides stercoralis* on agar plate culture

The plates were examined macroscopically and microscopically from the third day for the presence of the characteristic tracks and juvenile and adult stages of the parasite (Fig. 1). The surfaces of the plates were then washed with a formalin solution containing 10% formaldehyde, and the resulting solution was centrifuged at 1500 rpm for 5 min. The obtained sediments were examined using a light microscope for the presence of larvae or adult worms. The second-stage larvae (rhabditiform larvae) could be distinguished by their short buccal cavity, bulbous esophagus, and genital primordium. The third-stage larvae (filariform larvae) could be identified by their closed buccal cavity, long esophagus, and notched tail (Fig. 2) [21].

### Statistical analysis

All data were analyzed using the SPSS version 26.0 software package (IBM, Chicago, IL, USA). Chi-square (χ^2^) test or Fisher’s exact test was used for comparisons. In addition, Logistic regression models was used to determine Odds ratios (ORs) with 95% confidence intervals (CIs). A two-tailed *p* < 0.05 was considered statistically significant.

## Results

Of the 308 patients who participated in this study, 157 were admitted to a referral hospital for COVID-19 in Ahvaz, and the remaining patients were hospitalized in a hospital dedicated to COVID-19 in Abadan.

Patients enrolled in the study were aged 15–94, where the mean age of the participants was 57.99 ± 17.4 years. In addition, there were 156 (50.6%) male patients and 152 (49.4%) female patients. Furthermore, 12 patients (3.9%) had a positive ELISA test, 4 cases (1.3%) were equivocal, and 292 (94.8%) had negative ELISA tests. The ELISA test was repeated 2 to 4 weeks later in the four cases with equivocal results. However, because no rising antibody titer was found, it was deemed negative according to the kit protocol; nevertheless, parasitological tests were also performed, and all were negative (Table 1). Of the 12 ELISA-positive cases, three patients died prior to parasitological testing, three failed to provide stool samples, and only six underwent stool examinations. Two patients were found to be infected with *S. stercoralis* using both the direct smear examination and the APC method. However, only APC confirmed the presence of *S. stercoralis* infection in three cases (Table 2).

**Table 1:** Results of the enzyme-linked immunosorbent assay (ELISA) test of 308 COVID-19 patients participating in the present study

Characteristics	Groups	ELISA		*p*-value	OR (95% CI)
		Negative	Positive		
Gender	Female	150 (50.7%)	2 (16.7%)	0.037	5.137 (1.107–23.847)
	Male	146 (49.3%)	10 (83.3%)		
Age groups (years)	≤ 60	157 (53%)	2 (16.7%)	0.027	5.647 (1.216–26.218)
	> 60	139 (47%)	10 (83.3%)		
Location	Ahvaz	154 (52%)	3 (25%)	0.081	3.254 (0.864–12.257)
	Abadan	142 (48%)	9 (75%)

**Table 2:** Clinical data and results of parasitological examinations of 12 ELISA-positive strongyloidiasis cases

Case	Gender	County	Outcome	APC	DS	WBCs count/μL	Eosinophil (%)	Gastrointestinal symptoms
1	Female	Ahvaz	D	‒	‒	7400	2	Abdominal discomfort
2	Male	Ahvaz	D	‒	‒	13,600	3	Weight loss, diarrhea
3	Male	Ahvaz	D	‒	‒	19,800	2	Abdominal pain, nausea
4	Male	Abadan	I	Pos	Neg	13,100	2	Constipation, epigastric pain
5	Male	Abadan	I	Pos	Pos	5400	2	Weight loss, diarrhea, abdominal pain
6	Male	Abadan	I	‒	‒	8700	14	Nausea, anorexia, vomit
7	Male	Abadan	I	‒	‒	7400	7	Asymptomatic
8	Male	Abadan	I	‒	‒	7800	16	Asymptomatic
9	Male	Abadan	I	Pos	Pos	7600	27	Epigastric pain, intermittent diarrhea
10	Female	Abadan	I	Pos	Neg	5800	35	Abdominal pain, constipation
11	Male	Abadan	I	Neg	Neg	6600	8	Anorexia, epigastric pain
12	Male	Abadan	I	Pos	Neg	8500	22	Asymptomatic

*D* death, *I* improvement, *APC* agar plate culture, *Pos* positive, *Neg* negative, *DS* direct smear, *WBCs* white blood cells

In this study, there were statistically significant differences between *S. stercoralis* infection and sex (*p* = 0.037) and age (*p* = 0.027). However, there was no meaningful relationship between strongyloidiasis and the residence location of the patients (*p* = 0.081). Of the 12 cases with positive ELISA results, 9 (75.0%) resided in Abadan. The analysis confirmed the significant association between strongyloidiasis and sex (OR: 5.137; 95% CI 1.107–23.847), age (OR: 5.647; 95% CI 1.216–26.218), and location (OR: 3.254; 95% CI 0.864–12.257) (Table 1).

The five patients diagnosed with strongyloidiasis were administered 200 μg/kg/day ivermectin orally for 2 days, followed by another dose 2 weeks later [22]. In addition, they were followed up with APC and direct smear examination after one month, and all of their test results were negative.

## Discussion

Despite the strong emphasis on screening immunocompromised patients for asymptomatic strongyloidiasis due to the transformation of chronic strongyloidiasis to HS, clinicians in the clinic do not give this subject enough consideration, particularly in *S. stercoralis* endemic areas [1, 2].

During the coronavirus pandemic, the risk of HS increased, particularly in endemic regions, due to the increased use of tocilizumab and immunosuppressive drugs such as dexamethasone in COVID-19 patients [9, 14, 23]. Some evidence indicated that a dexamethasone dose of 6 mg/day for ten days (≈ 40 mg of prednisone) could induce HS in patients with chronic strongyloidiasis [24]. In addition, the accessibility and low cost of corticosteroids such as dexamethasone in most *S. stercoralis* endemic regions may increase the likelihood of this risk [24].

In the present study, a seroprevalence of 3.9% for strongyloidiasis underscores the significance and necessity of screening and treatment for co-infection with strongyloidiasis and COVID-19. Several cases of hyperinfection or disseminated infection in COVID-19-positive patients have been reported over the past 3 years. These severe conditions were observed during hospitalization or shortly after patients’ discharge [9, 25–27].

Strongyloidiasis is typically asymptomatic and challenging to diagnose using routine parasitological methods such as the direct smear method. Diagnostic methods with high sensitivity, particularly APC, are important when diagnosing patients; however, these methods are time-consuming and laborious and require fresh stool samples [21, 28]. In our study, the severity of gastrointestinal symptoms, specifically diarrhea, was observed in two patients due to an increasing parasite load and abundant larval excretion, which was easily detectable using the direct smear technique. However, in the remaining positive cases, only APC confirmed the presence of *S. stercoralis* infection.

The higher sensitivity and negative predictive value (NPV) of the ELISA test in detecting the mild form of strongyloidiasis, as well as the easier use of blood sampling for critically ill patients as opposed to stool sampling, have rendered the ELISA test a suitable method for screening for strongyloidiasis in COVID-19 patients [29].

The co-infection of nematode and virus is debatable in two additional ways: decreased eosinophil levels in the peripheral blood of COVID-19 patients and overlap of pulmonary and gastrointestinal symptoms during the acute phases of both infections [9, 30, 31].

In addition to its protective role in strongyloidiasis, eosinophilia is regarded as a screening factor with a high positive predictive value for asymptomatic patients. On the other hand, researchers recognize eosinopenia as a sign of the progression to severe cases of strongyloidiasis [28, 32–34]. Previous research indicates that eosinopenia is a common laboratory finding in 50–70% of hospitalized COVID-19 patients with mild to moderate symptoms [35, 36]. It is believed that the migration of eosinophils from the peripheral blood to the interstitial space and pulmonary alveoli, the decrease in the production of these cells, and their suppression by endogenous glucocorticoids are the leading causes of this decrease [37–39]. Some seropositive patients in the present study had a history of eosinophilia. Still, the rate of peripheral blood eosinophils in most of them had decreased significantly during hospitalization due to SARS-CoV-2 infection.

Corticosteroid therapy decreased the eosinophil count in a COVID-19 patient with a history of chronic hypereosinophilia due to strongyloidiasis, as reported by Stylemans et al. [40]. In addition, Feria et al. (2022) and Lier et al. (2020) found no increase in the number of peripheral blood eosinophils in patients co-infected with *Strongyloides* and COVID-19 [13, 14, 25].

Another attribute of strongyloidiasis-COVID-19 co-infection is the overlap of pulmonary and gastrointestinal manifestations caused by COVID-19 and those induced by HS, such as coughing, dyspnea, diarrhea, and fever [23]. In these instances, the physician will likely disregard the reactivation of *Strongyloides*. As a result, the patient is not referred to the parasitology laboratories and continues to take corticosteroids, which can exacerbate the infection [41].

Intriguingly, ten of the twelve positive ELISA cases involved patients older than 60. These findings are consistent with previous research indicating that a significant proportion of strongyloidiasis patients co-infected with COVID-19 are older adults [26, 27, 40, 42]. The higher prevalence of strongyloidiasis in elderly populations [43] can overlap with the higher COVID-19 mortality in these patients [44].

Similar to previous studies [45, 46] *S. stercoralis* infection was correlated with sex. The higher prevalence of infection in males may be attributable to prolonged contact with soil in occupations such as agriculture [47].

### Strengths and limitations

This study was one of the few studies conducted to screen strongyloidiasis in COVID-19 patients in an endemic region with serological and parasitological methods. The study had several limitations. First, the patient's medical records information could not be accessed. Second, the study was limited by the inability to conduct parasitological tests on some seropositive cases due to patient non-cooperation or death. Third, we were unable to evaluate the potential relationship between steroid dose and treatment of COVID-19 patients infected with *S. stercoralis*; therefore, future research is required.

## Conclusion

In conclusion, our findings demonstrated that screening for latent strongyloidiasis using high-sensitivity diagnostic methods, particularly ELISA, in COVID-19 patients before receiving suppressive drugs in endemic areas should be given greater consideration.

## Data Availability

All data analyzed during this study are included in this manuscript.

## Abbreviations

*S. stercoralis*: *Strongyloides stercoralis*
APC: Agar plate culture
CSS: Cytokine storm syndrome
SARS-CoV-2: Severe acute respiratory syndrome coronavirus 2
TCZ: Tocilizumab
ARDS: Acute Respiratory Distress Syndrome
ELISA: Enzyme-linked immunosorbent assay
NIE: Recombinant immunodiagnostic antigens
OD: Optical density
NTU: NovaTec units
NPV: Negative predictive value
